# Junaidi Space Regainer: A New Two-in-One Chairside Appliance to Simplify the Space Regaining Procedure

**DOI:** 10.7759/cureus.72518

**Published:** 2024-10-28

**Authors:** Mohammed Junaidi Dhahir

**Affiliations:** 1 Independent Researcher, Dr. Mohammed Junaidi Clinic, Erbil, IRQ

**Keywords:** 2-in-1 appliance, chairside, interceptive orthodontics, junaidi, new design, preventive orthodontics, space maintainer, space regainer

## Abstract

Premature loss of primary teeth can lead to crowding, rotation, ectopic eruption and impaction of permanent teeth. All of these issues can be reduced or avoided through proper preventive and interceptive orthodontic procedures including the use of space maintainers and space regainers, many of which require two visits and a laboratory phase for fabrication. The ‘Junaidi space regainer’ is an attempt to make the space regaining procedure simpler and easier. A 12-year-old boy presented to our clinic with a loss of space for the eruption of the second premolar; the Junaidi space regainer, which is composed of a band, a piece of archwire and an open coil spring, was used in this case. Following treatment, the space was successfully regained, allowing the second premolar to begin erupting into the regained space. The Junaidi space regainer is a two-in-one appliance that can serve both as a space regainer and a space maintainer. Its fabrication does not require taking impressions; in other words, a laboratory phase is not required. This new chairside space regainer provides a simple, time-saving and effective method for regaining lost space.

## Introduction

Interceptive orthodontic procedures are means by which a developing malocclusion can be eliminated or at least reduced in severity, along with the complexity, total treatment time, and cost of orthodontic treatment [1]. The most prevalent orthodontic concern is the insufficient arch space for the eruption of permanent teeth [2]. Effective management of space deficiencies resulting from the premature loss of primary teeth is a crucial aspect of both preventive and interceptive orthodontics [3].

Primary dentition plays a crucial role in guiding the eruption of permanent teeth, but many children lose these teeth prematurely as a consequence of dental trauma, neonatal tooth extraction, early childhood caries, or periodontal problems, or it can be a manifestation of systemic disease [4]. The most common causes of premature tooth loss are dental caries and trauma [5]. These also include ectopic eruption, congenital disorders, and early root resorption of primary teeth [3]. Many studies find that caries is the most common or one of the main causes of early loss of primary teeth [3,4,6-8]. Premature loss of primary teeth reduces the arch length [8,9] required for the succeeding tooth and, hence, predisposes teeth to crowding [2-5,8], rotation [8] and impaction and ectopic eruption of the permanent teeth [3-5], midline shift [3,8], and leads to malocclusion [2,3,5,7,8]. Premature tooth loss can also result in chewing difficulties [7]. Therefore, preservation of space is an integral part of preventive and interceptive orthodontics [4].

All kinds of space maintainers are frequently used in the maxillary and mandibular arches to assist in preserving the arch length after a primary tooth is extracted and to reduce the need for future orthodontic treatment. In cases where space is lost, space regainers can help restore it and avoid malocclusions, including crowding, that can happen later in dental development [10].

Conventional space regainers require taking an impression, laboratory procedures and more than one appointment [1]. Hence, this case report is aimed at reporting space regaining using the ‘Junaidi space regainer’, which was developed by the author. It is a new chairside appliance designed to regain the lost space and simplify the space regaining process.

## Case presentation

**Figure 1:**
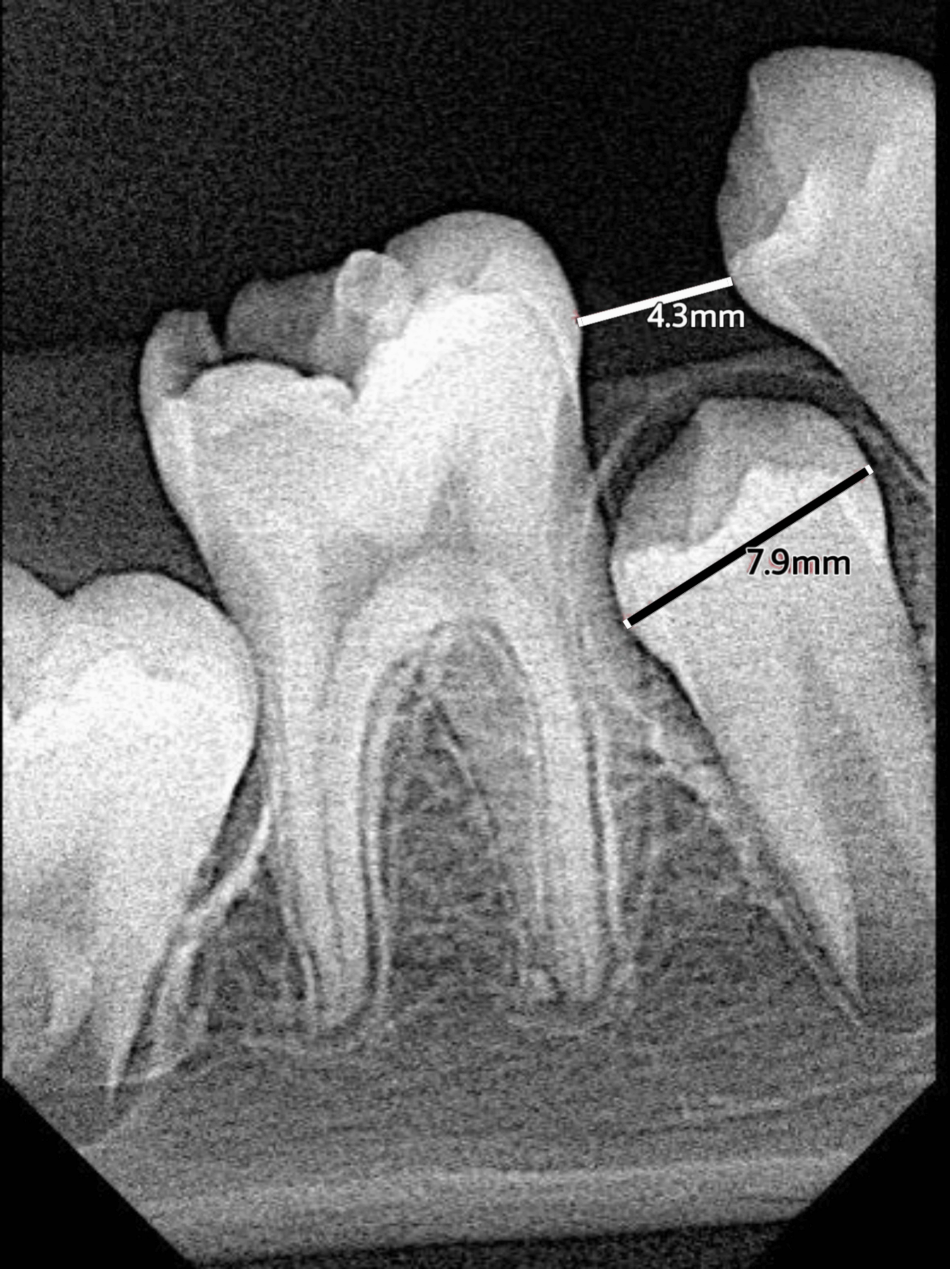
Pre-operative X-ray (available space 4.3 mm)

A 12-year-old male patient visited our clinic complaining of pain in the lower right permanent first molar. After the clinical and radiographic examinations, it was revealed that root canal treatment was indicated for that tooth. Meanwhile, a loss of space for the lower right second premolar was observed caused by the early loss of the lower right primary second molar (Figure 1). His parents were informed about the consequences and the necessity for space regaining. After discussing the interceptive option, they agreed to go ahead with the procedure, and informed consent was obtained.

**Figure 2:**
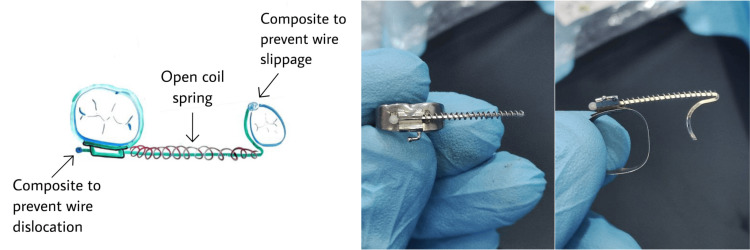
Design and fabrication of the Junaidi space regainer Image credit: Author's own

It was decided to regain the space and the Junaidi space regainer was used. All what we needed was a molar band, an orthodontic archwire and an open coil spring. The appropriate molar band size was selected and a large rectangular stainless steel wire (e.g., 0.018 x 0.025 inches) was measured and cut according to the distance between the first premolar and first permanent molar plus the space required, and the amount needed to bend it to accommodate the distal surface of the premolar. We can add 2 mm to that length for the composite ball that’s to be placed at the end of the wire distal to the tube of the molar band, or cinch back the wire. Before cementation, the molar band was fitted with the wire in place, and a nickel-titanium (NiTi) open coil spring was used; the length of the open coil spring was measured from the mesial opening of the molar band tube to the end of the wire arm, just before the wire turned back to accommodate the distal surface of the premolar, plus an additional 2-3 mm for activation. Then, the design was assembled and a ball-like piece of the flowable composite was attached to the wire end distal to the molar tube to prevent wire dislocation (Figure 2).

**Figure 3:**
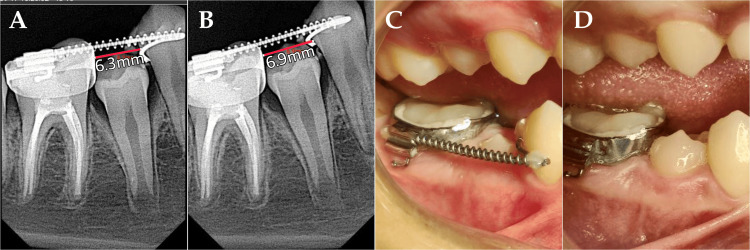
Follow-ups at (A) one month, space = 6.3 mm; (B, C) two months, space = 6.9 mm; (D) four months, space = 7.3 mm

Then the appliance was cemented and the other end of the wire was also attached to the premolar with the flowable composite, after etching and bonding to that area of the enamel, to prevent wire slippage. The patient was followed up and checked for any improvement (Figure 3). Finally, the space was regained successfully.

## Discussion

It is correctly stated that primary teeth serve as the best space maintainers for the permanent dentition [11]. The use of space maintainers can reduce the severity of problems such as crowding, ectopic eruption, tooth impaction and poor molar relationships [9]. Hence, space maintainers are used in preventive orthodontics [4]. A developing malocclusion can be intercepted to prevent it from progressing into a more complicated dentoskeletal problem [12]. That is why when space loss occurs, space regainers are required on a routine basis. Space regainers are the appliances that move the tooth mesially or distally to recover the lost space. These appliances help the permanent teeth erupt properly in their right place [3].

The extent of the problems associated with premature loss of primary teeth is not always the same. Studies indicate that when a primary second rather than a primary first molar is lost, or if tooth loss happens at an earlier age, or in crowded dentitions, greater space loss is expected [3] and premature loss of primary second molars clearly requires space maintenance [9].

The common methods of fabricating space regainers that are either removable or fixed appliances include a laboratory step and require two appointments for their fabrication and delivery. Appliances such as a banded helical space regainer and the sliding loop space regainer can also be used in such cases; however, the former requires soldering, while the latter requires a laboratory phase for fabrication.

Attempts have been made to make the fabrication of the appliances simpler, e.g. prefabricated appliances, which have a soldered wire to the band. This solder joint is a common site for the failure of the appliance, which after cement loss accounts for anywhere from 15% to 36% of failures [9]. The Junaidi space regainer does not use soldering and instead has a wire inserted into the tube of the band. The wire used is a large, heavy-gauge wire that can resist deformation better, and also acts as an anchor and a holding wire for the open coil spring. This wire can be easily adjusted; it can also be replaced with a new piece of wire, if needed, unlike laboratory-made rigid, soldered arms or wires. Hence, in case of any failure, the Junaidi space regainer can be fixed chairside, in contrast to the appliances that need a laboratory to be repaired or replaced.

For insertion, cement is applied to the band, and the appliance is pressed in a way that compresses the open coil spring, to aid in insertion. During follow-up appointments, the open coil spring can be reactivated by putting some flowable composite on the wire (Figure 3C) or by using a crimpable stop. After insertion, the mesial end of the wire is bonded to the premolar, or else the wire will move downward and impinge the gingiva during chewing, also making cleaning the area more difficult.

In addition to being easy to fabricate, it also reduces the number of appointments to a single appointment, and requires less time for its fabrication and delivery; it also does not require taking an impression and additional laboratory procedures. Moreover, it can be modified to serve as a space maintainer as well by a simple modification in the design, again done chairside. As it can serve two functions at the same time, as a space maintainer and space regainer, it is a two-in-one appliance. If space maintenance is indicated, it can be achieved by omitting the use of the open coil and placing a stopper mesial to the molar band tube, either through wire bending or the application of a crimpable stop. Additionally, once the appliance has been employed as a space regainer, it may be retained in situ without further modification, as the open coil is now passive.

## Conclusions

The Junaidi space regainer is a new two-in-one appliance that simplifies the space regaining process by being a simple, effective, and chairside appliance. In addition to being time-saving as it is made and delivered in a single visit, it can also function as a space maintainer by a simple change in the design. Additionally, the appliance can be fixed easily chairside, in case of any failure. Also, the appliance we use in our dental practice can be designed or modified to be multifunctional, to be used as a space maintainer and a space regainer.
